# Measuring a Critical Component of Contraceptive Decision Making: The Contraceptive Concerns and Beliefs Scale

**DOI:** 10.1007/s10995-023-03856-5

**Published:** 2024-01-09

**Authors:** Corinne H. Rocca, Isabel Muñoz, Lavanya Rao, Sara Levin, Ori Tzvieli, Cynthia C. Harper

**Affiliations:** 1 Advancing Standards in Reproductive Health (ANSIRH), Department of Obstetrics, Gynecology and Reproductive Sciences, School of Medicine, University of California, San Francisco, Oakland, CA USA; 2 Department of Epidemiology and Biostatistics, School of Public Health, University of California, Berkeley, Berkeley, CA USA; 3 Bixby Center for Global Reproductive Health, Department of Obstetrics, Gynecology and Reproductive Sciences, School of Medicine, University of California, San Francisco, San Francisco, CA USA; 4 Division of Public Health, Contra Costa Health, Martinez, CA USA

**Keywords:** Adolescent, Beliefs, Contraception, Family planning, Interpregnancy care, Psychometrics, Young adults

## Abstract

**Introduction:**

Concerns about safety and side effects from contraceptives are widespread and related to reluctance to use them. Measuring these concerns is an essential component of understanding contraceptive decision-making and guiding contraceptive and interpregnancy clinical care.

**Methods:**

We used qualitative research and item response theory to develop and test a psychometric instrument to measure contraceptive concerns and beliefs. We developed 55 candidate scale items and tested them among 572 adolescents and adults across nine California healthcare facilities in 2019–2020. We derived a 6-item scale and assessed differences by age and social determinants of health with multivariable regression.

**Results:**

In qualitative data, participants voiced both concerns and positive beliefs about contraception. Quantitative survey respondents were aged 21 years on average, and 24% were parous. Over half (54%) worried contraception has dangerous side effects, and 39% worried it is unnatural. The mean Contraceptive Concerns score, increasing with higher concerns, was 1.85 (SD: 1.00, range 0–4, α = 0.81). Items fit a partial credit item response model and met prespecified criteria for internal structure validity. Contraceptive use declined with increasing Concerns score (adjusted prevalence ratio [aPR] = 0.81 [0.72–0.92]). Scores were elevated among Black (mean: 2.06; aβ = 0.34 [0.09, 0.59]) and Multiracial or other race (2.11; aβ = 0.34 [0.02, 0.66]) respondents vs. White (1.66), but not Latinx respondents (1.81; aβ = 0.11 [− 0.11, 0.33]). Scores were also elevated among participants with lower maternal education (high school/Associate’s 1.89 versus college 1.60; aβ = 0.28 [0.04, 0.53]).

**Discussion:**

The psychometrically robust Concerns instrument can be used in research to measure autonomous contraceptive decision-making and to design person-centered care.

## Significance

**What is already known on this subject?:**

Concerns about safety and side effects from contraceptives are prevalent and must be considered when designing and evaluating contraceptive care, including in the interpregnancy period. However, to date no rigorously developed instrument has existed.

**What this study adds?:**

The six-item Contraceptive Concerns instrument is psychometrically robust and can be used in research to measure and understand autonomous contraceptive decision-making and to design equitable person-centered contraceptive care.

## Introduction

People’s beliefs about and acceptability of contraceptives, including hormonal and long-acting methods, are essential considerations for their method preferences and use (Alspaugh et al., 2020; Gilliam et al., 2009; Guendelman et al., 2000; Le Guen et al., 2021). Skepticism about method safety, including suspecting that hormones are harmful, cause cancer, or that users need to take periodic breaks, are commonly cited reasons for reluctance to use contraception in the United States (Guzzo & Hayford, 2018; Rocca & Harper, 2012). Worries about potential emotional and physical side effects and beliefs that methods are unnatural are also prevalent (Littlejohn, 2013; Woodsong et al., 2004). Concern about side effects is a top reason for not using contraception (Frederiksen & Ahrens, 2020) and a primary reason for discontinuing reversible prescription methods (Moreau et al., 2007). Negative perceptions about methods can stem from structural inequities and coercive promotion of contraception targeting low-income communities and communities of color, which have led to mistrust of pharmaceutical companies, the medical system, healthcare providers, and researchers (Rocca & Harper, 2012; Thorburn & Bogart, 2005; Woodsong et al., 2004). Skepticism about contraceptives has also been documented in communities that have lower health literacy, language barriers or less communication about reproductive health (Grossman et al., 2010; Guendelman et al., 2000).

While concerns and beliefs come into play in decisions about contraception, the reproductive health field has remained without standardized measures. Most large-scale datasets and studies assessing contraception, including the National Survey of Family Growth (Frederiksen & Ahrens, 2020), National Longitudinal Study of Adolescent to Adult Health (Guzzo & Hayford, 2018), and the National Survey of Reproductive and Contraceptive Knowledge (Rocca & Harper, 2012), among others (Callegari et al., 2017; Cramer & DeRoy, 2019; Rocca et al., 2022), have employed individual questions to assess facets of concerns about contraception. These studies have asked whether using contraception is too onerous, or whether a severe mood problem is likely to result from pill use, for instance. However, the psychometric performance of a multi-item scale has not yet been formally evaluated against established criteria for reliability and validity (Reeve et al., 2007; Wilson, 2006), which makes it difficult to compare results across studies. More importantly, a measure is needed to prioritize findings for development of contraceptive programs and policies to meet people’s needs and preferences.

In 2021, contraceptive policy recommendations highlighted an urgent need to develop new measurement tools that ensure the delivery of just, person-centered care that upholds reproductive autonomy (Coalition to Expand Contraceptive Access, 2021). An easy-to-use instrument that captures people’s concerns and beliefs about contraception is an important step toward understanding their contraceptive preferences, desires, and autonomous use. In this mixed methods study, we addressed this gap by developing a psychometrically robust instrument, the Contraceptive Concerns and Beliefs Scale (Concerns Scale for short), based on items from qualitative data and the scientific literature. We then examined how concerns and beliefs differed among sociodemographic groups to further our understanding of long-standing differences in method use and undesired pregnancy by age, race and ethnicity, and socioeconomic status.

## Methods

Our scale development process was comprised of a literature review and qualitative research, delineation of conceptual domains and definitions, item development, quantitative field testing, and psychometric analysis (Wilson, 2006). Building on prior contraceptive research (Raine-Bennett & Rocca, 2015), the work was guided by the Necessity-Concerns Framework from medication adherence research (Horne et al., 2013). This framework postulates that, when determining whether to initiate or continue using a medication, individuals balance their perceived need for the medication (necessity) with their concerns and the perceived benefits of using it (concerns). Applied to prescription contraception, we view individuals’ willingness to use contraception as being driven not only by desire to avoid pregnancy (necessity), but also consideration of pertinent concerns and benefits about contraception, including its acceptability and worry about safety and side effects (concerns). Our work aims to develop a measure capturing the construct of contraceptive concerns and beliefs.

### Qualitative Research

Our first step was to conduct a rigorous literature review and original qualitative research to inform the development and wording of scale items (Muñoz et al., 2020). Prior to data collection, the key content areas and topic guides were reviewed by the community advisory board of the University of California, San Francisco (UCSF) Preterm Birth Initiative. We then held seven focus group discussions (FGDs, N = 42) and 13 in-depth interviews (IDIs) among 55 sexually active adolescents and adults aged 15–29 years recruited from three reproductive health clinics in Northern California. Participants had to be assigned female at birth due to our interest in beliefs and concerns about prescription contraceptives, which are only available for these individuals. FGDs were scheduled for particular times, and those who could attend were asked to, while IDIs were scheduled for other participants. Trained focus group moderators and interviewers asked participants about their lived experiences with contraception, their likes and dislikes about contraceptive methods, and the impact contraception has had on their bodies and lives. FGDs were age-stratified (adolescents aged 15–19 years, adults aged 20–29); all participants identified as cisgender. Childcare was provided, and participants were remunerated $100 for FGD participation (about 75 min) and $50 for interview participation (about 45 min). We continued data collection until we reached thematic saturation.

One-quarter of qualitative research participants identified as Latinx; 26% as White; 19% as Black; 15% as Asian, Pacific Islander, Hawaiian Native; and 15% as Multiracial or other races. Participants voiced both negative and positive attitudes about contraception from their own experiences and those of their peers (Muñoz et al., 2020). Of primacy were frustrations with side effects, including moodiness and irregular bleeding, and feelings that contraception is “invasive” or “not natural.” Participants expressed skepticism about long-term safety and fears about future fertility. Views on contraception reached beyond their own bodies and were situated within a social context of family member and community judgement (e.g., “looked down upon”). Participants also verbalized benefits of using contraception across similar domains, including favorable side effects (e.g., “controls the cramping”), the benefits of preventing pregnancy (“peace of mind”), and positive social perceptions (“being responsible”).

### Domains and Item Development

We originally adopted a broad working definition of the construct we aimed to measure, conceptualizing “contraceptive acceptability” as a person’s feelings and opinions that they accept to be true about prescription contraceptive methods. Based on the qualitative work and existing literature, we identified seven interrelated draft domains, each comprised of the negative aspects or concerns and positive aspects or benefits. The primary domains referenced safety, health and side effects from contraceptive use, as well as skepticism and trust around the promotion of contraception. Other domains included concerns with the process of obtaining and using contraception and stigma, and benefits including pregnancy prevention and positive connotations of use (Appendix). We did not know at the outset if the construct was unidimensional, if the draft domains themselves would comprise unique psychometric dimensions, or if the negative and positive aspects would fall into separate dimensions.

Based on the conceptual domains, we developed a library of candidate items for the measure, with each item borne directly from our qualitative work or published peer-reviewed qualitative literature. Items were translated into Spanish, and we honed items and their translations based on feedback from ten cognitive interviews with additional individuals from the same patient populations from whom we drew the focus groups and interviews. The final 55 candidate items covered the seven domains; each item was a statement about contraception, to which respondents indicated if they agreed, somewhat agreed, neither agreed nor disagreed, somewhat disagreed, or disagreed. Items were coded so that higher levels of concerns were higher on a 0–4 scale.

Initial psychometric analyses based on the large field test (see below) indicated that our original conceptualization was too broad a construct to measure with a single measurement instrument. We thus refined our construct to focus more narrowly on side effects, health and safety, skepticism and trust and named the scale the Concerns Scale for short. Related aspects of contraceptive acceptability, including concerns with obtaining and using contraception and stigma, were not included.

### Quantitative Field Test

We tested the 55 items among patients seeking reproductive care from nine reproductive and primary care health facilities in the San Francisco Bay area between June 13, 2019 and February 26, 2020 (Harper et al., 2022). Study facilities were primarily Department of Health and non-profit community clinics, including Federally Qualified Health Centers, School-based Health Centers, reproductive health clinics, and an outpatient public hospital obstetrics and gynecology clinic. Trained bilingual research assistants (RAs) approached all individuals in the waiting room and described the study; the RA was English/Spanish bilingual 80% of recruitment time. To participate, patients had to be aged 15–34 years, assigned female at birth, sexually active in the prior six months, and able to read and speak English or Spanish. The RA obtained verbal consent to participate from eligible and interested patients using a tablet. Participants then completed the 30-min anonymous electronic survey on the same device in the waiting room. Contraceptive Concerns items were early in the survey to facilitate their completion prior to the clinical appointment, but the few participants who were called into their appointment could complete the survey afterwards. Participants received $20 cash or gift card upon survey completion. The study was approved by UCSF’s Institutional Review Board and was performed in accordance with the ethical guidelines of the 1964 Declaration of Helsinki.

### Psychometric Analysis

We used item response theory (IRT) for analyses (De Boeck & Wilson, 2004; Hays et al., 2000), supplemented with exploratory factor analysis and classical methods to determine dimensionality and internal consistency (Cronbach, 1990). Our aim was to iteratively reduce the 55 items to a set of 4–6 items, given it is rarely feasible to include long scales in contraceptive research surveys. Our approach balanced creating a scale with high internal consistency (i.e., reliability) while also capturing the full scope of contraceptive concerns (i.e., validity).

To select scale items, we first assessed item completion, removing those with > 5% missing. We examined the distribution of responses to each item to ensure items served to differentiate respondents’ attitudes and removed those with highly skewed responses, as they did little to differentiate respondents’ concerns levels (Edelen & Reeve, 2007).

Using ACER ConQuest 4.5 (Camberwell, Australia), we iteratively fit item responses to unidimensional partial credit item response models and examined item fit, internal structure validity, and differential item functioning, removing less optimally performing items until we arrived at six final items. We considered a weighted mean-squared index of 0.75–1.33 as indicative of good fit to the model (Wright & Masters, 1982). For internal structure, we ensured that for each item, respondents endorsing higher response categories (reflecting greater concerns) had correspondingly higher overall Concerns scores. We also plotted item thresholds relative to attitudes levels (e.g., Wright Maps) to ensure items served to differentiate respondents along the spectrum of attitudes and confirm correct ordering of each item’s category locations.

Once the six items were selected, we fit a final series of models to establish item parameters and the scale’s psychometric properties. We repeated the steps outlined above and assessed internal consistency with the separation reliability coefficient. We fit four new partial credit differential item functioning (DIF) models, each which incorporated item-by-characteristic interaction terms (De Boeck & Wilson, 2004). The characteristics included age, sexual orientation, race and ethnicity, and maternal education level as an indication of socioeconomic status (SES). We used maternal education as a socioeconomic indicator rather than the participants’ educational level because almost half of the sample was adolescent and still pursuing additional education, and many were unlikely to know their household incomes. We considered item-by-characteristic parameter effect sizes of ≥ 0.6 logits as evidence of DIF (Paek, 2002; Steinberg & Thissen, 2006).

Supplementing the IRT analyses, we used exploratory factor analysis to ensure the scale’s items loaded onto a single factor with eigenvalue > 1 (Kline, 1986). We averaged summed raw scores across items, calculating item-total correlations and examining internal consistency (Cronbach’s α).

Finally, as a test of external validity, we investigated the relationship between Concerns scores and current use of a prescription contraceptive method, fitting a Poisson regression model and calculating predicted probabilities of use. We also fit a multivariable model, using multiple imputation with chained equations to account for missing covariable data. We hypothesized that higher Concerns scores would be associated with lower contraceptive use.

Using the final Concerns Scale, we investigated variations by sociodemographic characteristics with bivariable linear regression and fit a multivariable model using multiple imputation for missing data. We used Stata 15 for classical and regression analyses (College Station, TX).

## Results

A total of 580 respondents enrolled into the study. Among the 580, eight were called into their clinical appointments before completing the Concerns Scale items and did not return, leaving 572 in the analysis sample. They were 21 years of age on average; 43% of the sample was aged 15–19 and 30% aged 20–24 years (Table 1). Thirty-four percent identified as primarily Latinx; 25% as White; 20% as Black; 11% as Asian, Pacific Islander, or Hawaiian Native; and 10% as Multiracial or other races. Over two-thirds (76%) were nulliparous. Nine percent were married, and 84% overall had a main romantic partner. While 84% were heterosexual or mostly heterosexual, 14% were bisexual and 2% mostly gay/lesbian, pansexual or other. All reported being cisgender. Participants most commonly reported using condoms (36%), followed by withdrawal/fertility awareness (19%), injectables (15%) and oral contraceptive pills (14%). Among the 49% reporting use of a prescription method, 41% also reported condom use, withdrawal/fertility awareness, and emergency contraception.

**Table 1:** Respondent characteristics (n = 572)

	*n*	*%*
*Age,* mean years, SD (range:15–33) (n = 559)	21.3	4.6
*Age group (n = 559)*		
15–19 years	239	42.8
20–24 years	167	29.9
25–33 years	153	27.4
*Race and Ethnicity*		
Latinx	193	33.7
White	144	25.2
Black	112	19.6
Asian, Pacific Islander, or Hawaiian Native	64	11.2
Multiracial or other	59	10.3
*Maternal education* (n = 542)		
Less than high school	188	34.7
High school, GED, Associate’s, vocational	271	50.0
College degree or more	83	15.3
*Parity* (n = 555)		
0	422	76.0
1	77	13.9
2 or more	56	10.1
*Married* (n = 565)	53	9.4
*Has a main/serious partner* (n = 565)	477	84.4
*Had sex in the last month* (n = 548)	472	86.1
*Sexual orientation* (n = 558)		
Heterosexual	387	69.4
Mostly heterosexual	80	14.3
Bisexual	79	14.2
Mostly gay/lesbian	4	0.7
Pansexual or Other	8	1.4
*Primary reason for clinic visit* (n = 571)		
Contraceptive care	257	45.0
STI testing	117	20.5
Pregnancy test, pre/postnatal	97	17.0
Annual, illness, non-reproductive, other	100	17.5
*Contraceptive methods used in last 30 days* ^a^ (n = 562)		
Condom	203	36.1
Withdrawal or fertility awareness ^b^	107	19.0
Depo-Provera (injectable)	82	14.6
Oral contraceptive pill	80	14.2
Implant	57	10.1
Intrauterine device (IUD) ^c^	45	8.0
Emergency contraception	26	4.6
Transdermal patch	19	3.4
Vaginal ring	8	1.4
None	136	24.2

^a^ Respondents could indicate use of more than one method. Among those reporting use of a prescription method (injectable, pill, implant, IUD, patch, or ring), 41% also reported use of condoms, withdrawal or fertility awareness, or emergency contraception

^b^ Withdrawal n = 104; Fertility awareness method n = 3

^c^ Includes n = 2 reporting sterilization

### Item Reduction

In iterative reduction of the item set, items that referred to broad concerns or were generally applicable (e.g., “Birth control affects my body in ways that I do not like”) functioned well across the full sample. In contrast, items referring more narrowly to specific concerns (e.g., “I worry that using birth control lowers my sex drive”) tended not to meet statistical criteria. Most items addressing the benefits of contraception, including all items in the pregnancy prevention and life benefits domains, were dropped because there were so many positive responses that they did not serve to distinguish respondents from each other. For instance, a full 88% agreed or somewhat agreed that “using birth control helps me have more control over my life.” Items related to concerns about the process of getting and using contraception (e.g., “I find birth control confusing to use”) and stigma of use (e.g., “I worry that using birth control makes it seem like I am sleeping with a lot of people”) appeared to represent different constructs than items in our primary domains and thus were dropped from this instrument.

We selected six items for the final Concerns Scale, including four negative and two positive items. Responses to items reflected the range of concerns and beliefs about contraception (Table 2). For example, 52% agreed or somewhat agreed that using birth control is good for their health and wellbeing, and 44% agreed that they believe the hormones in birth control are safe. However, 54% worried birth control has dangerous side effects, 38% that birth control affects their body in ways they do not like, and 42% worried drug companies hide information about birth control from women.

**Table 2:** Concerns Scale items and frequencies

	*n*	*%*
*Using birth control is good for my health and wellbeing.* (+ , Health and Safety) (n = 570)		
Agree	165	29.0
Somewhat agree	129	22.6
Neither agree nor disagree	185	32.5
Somewhat disagree	44	7.7
Disagree	47	8.3
*I worry that birth control has dangerous side effects.* (–, Side effects) (n = 568)		
Disagree	121	21.3
Somewhat disagree	48	8.5
Neither agree nor disagree	95	16.7
Somewhat agree	175	30.8
Agree	129	22.7
*I worry that using birth control is unnatural.* (–, Health and Safety) (n = 567)		
Disagree	190	33.5
Somewhat disagree	44	7.8
Neither agree nor disagree	113	19.9
Somewhat agree	132	23.3
Agree	88	15.5
*I believe the hormones in birth control are safe*. (+, Health and Safety) (n = 571)		
Agree	121	21.2
Somewhat agree	130	22.8
Neither agree nor disagree	174	30.5
Somewhat disagree	96	16.8
Disagree	50	8.8
*Birth control affects my body in ways that I do not like*. (–, Side effects) (n = 566)		
Disagree	173	30.6
Somewhat disagree	34	6.0
Neither agree nor disagree	139	24.6
Somewhat agree	124	21.9
Agree	96	17.0
*I worry that drug companies hide information about birth control from women* (–, Skepticism/Trust) (n = 570)		
Disagree	138	24.2
Somewhat disagree	44	7.7
Neither agree nor disagree	148	26.0
Somewhat agree	131	23.0
Agree	109	19.1

(+): Item coded so that “agree” = 0, “somewhat agree” = 1, “neither” = 2, “somewhat disagree” = 3, and “disagree” = 4.

(–): Item coded so that “disagree” = 0, “somewhat disagree” = 1, “neither” = 2, “somewhat agree” = 3, and “agree” = 4

### Psychometric Properties

Combined into a scale, scores on the Concerns Scale covered the full 0–4 score range (mean: 1.85, SD: 1.00, higher scores indicating higher level of concern) with a fairly symmetrical distribution. The six items had item-total correlations ranging from 0.62 to 0.80, with a Cronbach’s α of 0.81, and they loaded on to a single factor with an eigenvalue > 1 (Table 3). Fit to a unidimensional partial credit model, all items demonstrated good model fit (weighted mean square fit statistics 0.87 to 1.11). Item difficulty parameters ranged from − 0.33 to 0.34 logits. All items met prespecified criteria for internal structure validity, including that each item had response categories that corresponded to overall scores.

**Table 3:** Concerns Scale reliability and item properties (n = 572)^a^

	Classical Test (Cronbach’s α: 0.81)		Item Response Model (Person separation reliability: 0.73)	
	Item-Total correl	Factor loading	Model fit^b^	Difficulty^b^
Good for health and wellbeing ( +)	0.62	0.52	1.11	0.34
Worry dangerous side effects (–)	0.80	0.75	0.87	− 0.33
Worry unnatural (–)	0.76	0.69	0.96	0.05
Believe hormones are safe ( +)	0.68	0.59	1.01	0.14
Affects body in ways don’t like (–)	0.72	0.62	1.06	− 0.03
﻿Worry drug companies hide (–)	0.70	0.61	1.10	− 0.17

^a^ Nine respondents were missing data on one to four of the six items. For both psychometric approaches, scale scores were calculated based on non-missing item responses

^b^ Model fit is the weighted mean-squared fit t-statistic. Item difficulty is the difficulty parameter in logits. Model fit and difficulty are from a unidimensional partial credit item response model for polytomous items

( +): Item coded so that “agree” = 0 and “disagree” = 4

(–): Item coded so that “disagree” = 0 and “agree” = 4

The Concerns Scale items demonstrated excellent external validity. Tests for differential item functioning found no evidence for any item by age, race and ethnicity, sexual orientation, or maternal education level. In a multivariable Poisson model, probability of using a prescription contraceptive method decreased about 19% for each point increase in Concerns score (adjusted prevalence ratio [aPR] = 0.81, 95% CI 0.72–0.92, p ≤ 0.001). While a predicted 70% of respondents with a Concerns score of 0 used a prescription contraceptive, 30% of those with a score of 4 did. The only sociodemographic variable associated with contraceptive use was having a main partner (aPR = 1.66, 95% CI 1.09–2.52, p < 0.05; not shown).

### Associated Factors

Turning to sociodemographic differences in overall scores, adolescents had the lowest Concerns scores (mean: 1.67). In the multivariable model, scores were significantly lower among adolescents than those aged 25–33 years (aβ = − 0.26, 95% CI − 0.50, − 0.03, p < 0.05) (Table 4). Scores also differed by race and ethnicity: Black (mean: 2.06) and Multiracial or other race (mean: 2.11) respondents had the highest scores, while Latinx (mean: 1.81) and White (1.66) respondents had the lowest. These scores differed significantly in the multivariable model (Black vs. White: aβ = 0.34, 95% CI 0.09, 0.59, p < 0.05; Black vs. Latinx: p < 0.05). Regarding SES, respondents whose mothers had a high school, GED, Associate’s, or vocational degree (mean: 1.89) had higher Concerns scores than those whose mothers had completed a college degree or more education (mean: 1.60; aβ = 0.28, 95% CI 0.04, 0.53, p < 0.05).

**Table 4:** Mean Concerns Scale scores by respondent characteristics and β coefficients from a multivariable linear regression model^a^ predicting Concerns scores (range 0–4) (n = 564)

	Mean Score (SD)	Multivariable Model^b^ aβ Coefficient (95% CI)	
Total	1.85 (0.99)		
*Age group*			
15–19 years	1.67* (0.90)	− 0.26* (− 0.50, − 0.03)	
20–24 years	1.93 (1.05)	− 0.10 (− 0.32, 0.12)	
25–33 years (reference)	2.00 (1.00)	–	
*Race and Ethnicity*			
Latinx	1.81 (0.94)	0.11 (− 0.11, 0.33)	
White (reference)	1.66 (1.04)	–	
Black	2.06**^c^** (1.00)**	0.34**^c^** (0.09, 0.59)**	
Asian, Pacific Islander, Hawaiian Native	1.84 (0.87)	0.18 (− 0.11, 0.47)	
Multiracial/other	2.11** (1.06)	0.34* (0.02, 0.66)	
*Maternal education*			
Less than high school	1.84 (0.99)	0.26 (0, 0.53)	
High school, GED, Associate’s, vocational	1.89* (0.97)	0.28* (0.04, 0.53)	
College degree or more (reference)	1.60 (1.06)	–	
*Parity*			
Nulliparous (reference)	1.81 (0.97)	–	
Parous	1.95 (1.02)	− 0.02 (− 0.24, 0.21)	
*Has a main/serious partner*			
No (reference)	1.81 (0.93)	–	
Yes	1.85 (1.00)	0.09 (− 0.14, 0.31)	

***p ≤ 0.001, **p ≤ 0.01, *p < 0.05

^**a**^All models control for recruitment site

^b^Multivariable model fit using multiple imputation

^c^Black differs from Latinx at p < 0.05 (bivariable and multivariable)

## Discussion

We developed and assessed a six-item instrument to measure concerns and beliefs about contraceptives. About half of respondents agreed that contraception was good for their health and wellbeing. Still, over half worried that it has dangerous side effects, and two-fifths were skeptical about whether drug companies share all information about contraceptives. The final scale items fit a unidimensional item response model, were internally consistent, and had excellent internal structure. Individual items functioned non-differentially across respondent characteristics, and overall scale scores were highly correlated with prescription method use, findings demonstrating strong external validity.

Concerns around contraceptives were higher among those with lower maternal education and among Black and Multiracial or other respondents compared to Latinx and White respondents. These findings are consistent with prior scientific research (Rocca & Harper, 2012; Rosenthal & Lobel, 2020) and likely reflective of broad, long-standing reproductive health inequities and racism (Chambers et al., 2021; Roberts, 1997). Efforts to control the fertility and reproduction of Black and other women of color have led to negative beliefs about the motivations of the government and medical establishment in promoting contraception, related to genocide and contraceptive safety (Roberts, 1997; Thorburn & Bogart, 2005). Negative experiences in reproductive healthcare settings, including interactions with culturally incompetent providers, as well as experiences of friends and family, influence contraceptive beliefs and choices (Chambers et al., 2021; Gomez et al., 2020). Littlejohn has found that women interpret the seriousness of their own experiences with contraceptive side effects within the norms of their communities (Littlejohn, 2013). Perceptions of side effects are thus likely imbued with social group meanings, informed by legacies of discrimination and medical mistrust. Explicitly integrating an antiracist lens and awareness of historical and current inequities into curricula for providers may improve patient-provider trust, quality of contraceptive care, and ultimate contraceptive satisfaction among people of color and lower socioeconomic status.

In our sample, adolescents expressed lower levels of contraceptive concerns compared to adults. Prior research has demonstrated how contraceptive decision-making is dynamic and evolves over the life course, with individuals reevaluating their choices based on past experiences (Downey et al., 2017; Gomez et al., 2020). Given the strong experiential basis of contraceptive beliefs, the age-related increase may be due to greater experience with different methods over time. It is also possible that, as people become more open to pregnancy with age (Samari et al., 2020), the drawbacks of contraceptive use begin to outweigh the benefits (Horne et al., 2013). Contraceptive counseling that acknowledges potential side effects and invites people to change methods may increase method satisfaction and reduce concerns over the life course (Dehlendorf et al., 2014; Schivone & Glish, 2017).

This research has limitations. Our goal was to create an instrument to capture concerns and beliefs about contraceptives to ensure broad applicability in research. Certain items, including aspects of stigma, did not work well with scale items. Similarly, research has shown fear about future infertility is prevalent; the item we tested regarding this concern did not meet psychometric criteria for validity for our scale, suggesting that infertility concerns comprise a related but different construct than our measure captures. We also suspect that the relevance of fear around infertility is affected by pregnancy preferences, but we lacked a measure of pregnancy preferences. Future research should examine the interplay between pregnancy preferences and beliefs about contraceptives. Our analyses provide evidence that the Concerns Scale is psychometrically robust for research purposes; additional work will be needed to determine how the instrument can best be leveraged to improve clinical care. Finally, although this work was conducted with racially and ethnically diverse individuals across different types of reproductive and primary care community clinics, respondents were aged 15–33 years and seeking healthcare and cisgender. Additional testing of the items is needed in non-clinical samples, older individuals, geographic regions other than California, and globally (Senderowicz & Maloney, 2022).

## Conclusion

Efforts to help people to engage in contraceptive behavior that aligns with their reproductive preferences, including for birth spacing, have typically focused on improving contraceptive knowledge and access (Bharadwaj et al., 2012). While these factors remain critical, concerns about safety and side effects are prevalent and must also be considered when designing and evaluating equitable and person-centered contraceptive and interpregnancy care. Delivering care that upholds reproductive autonomy requires understanding and respecting individuals’ contraceptive needs and desires, including their concerns. The psychometrically robust Concerns instrument can be used in research to measure autonomous contraceptive decision-making and to design person-centered care.

## Data Availability

Not applicable.

## Appendix: Item Library for the Development of the Contraceptive Concerns Scale, by Domain

The Appendix shows the conceptual domains of the Contraceptive Concerns Scale, the 55 items developed and considered for inclusion in the scale, and the six final scale items (bolded). Some items can reasonably be placed across more than one domain.

**Table:**

Domain	Library of Items for the Contraceptive Concerns Scale
Side Effects	**- Birth control affects my body in ways that I do not like** **- I worry that birth control has dangerous side effects** - Birth control affects my emotions in ways that I don’t like - I worry that using birth control makes me gain weight - [I feel that] using birth control gives me painful cramps - I worry that using birth control lowers my sex drive - I feel that using birth control makes me have food cravings + I feel that using birth control makes my periods more regular + Birth control affects my body in ways that I like + Birth control affects my emotions in ways that I like + I’m willing to put up with the side effects of birth control so that I don’t get pregnant
Health and Safety	**- I worry that using birth control is unnatural** - I worry that the hormones in birth control are dangerous - I worry that birth control is actually unsafe - Birth control has long term effects on my health that I don’t like - I worry about hormones staying in my body after I stop using birth control - I worry that using birth control will make it harder for me to get pregnant when I want to - I worry that using birth control makes it more likely that I will get cancer - The idea of having hormones from birth control inside my body bothers me - [I worry that] birth control is invasive for my body ** + Using birth control is good for my health and wellbeing** ** + I believe the hormones in birth control are safe** + Using birth control is good for my health + I am ok with having hormones from birth control in my body
Skepticism/ Trust	**- I worry that drug companies hide information about birth control from women** - I feel that clinics push birth control to keep people like me from + having children - I worry that drug companies care more about making money than about making sure birth control is safe + I feel that drug companies make sure that women know about the side effects of birth control + Drug companies make sure birth control is safe
Process of obtaining and using^a^	- It is hard for me to find a place to get birth control - I feel that getting birth control can be embarrassing - Getting birth control is too difficult for me - I find birth control confusing to use - Using birth control is embarrassing for me
Pregnancy Prevention^b^	+ Using birth control does a good job of protecting me against pregnancy + I am happy that I don’t have to worry about becoming pregnant with birth control + Using birth control allows me to make my own decisions about becoming pregnant
Stigma^a^	- I worry that my sexual partner(s) will judge me for using birth control - I worry that my family will judge me for using birth control - I worry about my privacy when I get birth control - Using birth control makes me feel guilty - Using birth control goes against my morals - I worry that my sexual partner(s) will think I’m sleeping with someone else if I’m on birth control - I worry that people I know will look down on me for using birth control - I worry that using birth control makes it seem like I am sleeping with a lot of people - I worry that my sexual partner(s) will think I have an STD or a disease if I use birth control - Using birth control complicates my relationship with my family
Life benefits and positive implications^b^	+ Using birth control helps me have more control over my life + I feel that using birth control lets me focus on the things I want to do in my life + Using birth control makes me feel independent + Using birth control lets me be more active + Using birth control makes me feel like I am taking care of myself + I feel empowered when I use birth control + Using birth control makes me feel like I am in control of my own body + I feel that using birth control lets me be more “in the moment” when I have sex

^a^ Items in the process of obtaining and using stigma domains were excluded from the final scale because they appeared to represent different constructs.

^b^ Items in the pregnancy prevention and life benefits and positive implications domains were excluded from the final scale because respondents overwhelmingly agreed with these items, so they did not serve to distinguish respondents.
